# Object Exploration and Manipulation in Infants at Typical vs. Elevated Likelihood for ASD: A Review

**DOI:** 10.3390/children11070825

**Published:** 2024-07-05

**Authors:** Valentina Focaroli, Fabrizio Taffoni, Andrea Velardi, Barbara Caravale, Flavio Keller

**Affiliations:** 1 Department of Economic, Psychological and Communication Sciences, Niccolò Cusano University, 00166 Rome, Italy; 2 CREO LAB, Advanced Robotics and Human Centred-Technologies Laboratory, Campus Bio-Medico University, 00128 Rome, Italy; f.taffoni@unicampus.it; 3 Department of Humanities, Motor Sciences and Education, Niccolò Cusano University, 00166 Rome, Italy; andrea.velardi@unicusano.it; 4 Department of Developmental and Social Psychology, Sapienza University, 00185 Rome, Italy; barbara.caravale@uniroma1.it; 5 Laboratory of Developmental Neuroscience, Campus Bio-Medico University, 00128 Rome, Italy; f.keller@unicampus.it

**Keywords:** elevated likelihood for autism spectrum disorder, fine motor skills, manipulation, motor planning

## Abstract

The present review considers the growing body of literature on fine motor skills in infants at elevated genetic likelihood (EL) for autism spectrum disorder (ASD). This area of study aims to identify crucial motor markers associated with the disorder, facilitating earlier and more accurate identification of ASD, using various experimental methodologies, including standardized assessments, observational measures, and technological tools. The reviewed evidence revealed distinct developmental trajectories in EL infants, marked by differences in fine motor skills and exploratory behaviors compared to typically developing infants. We discuss the developmental trajectory of fine motor skills in infants and their predictive value for later ASD diagnosis, highlighting the significance of fine motor skills as early indicators of ASD risk in infants and emphasizing the need for further research to elucidate their predictive value and underlying mechanisms.

## 1. Introduction

Over the last decades, the development of motor skills has received renewed interest in the field of developmental science. This might be at least partially explained by the generalized importance that the sensorimotor system has in child development: many studies have evidenced the influence that the acquisition of motor skills can have on other developmental domains, beyond the motor one (e.g., [1,2,3,4,5]). This robust body of literature indicates that advancements in the motor domain enable children to encounter a wider range of opportunities for developing, practicing, and refining skills across various areas, especially in language and social interaction [6]. 

Among the wide repertoire of motor skills that emerge in early development, the ability to manipulate objects is considered a critical milestone. Reaching and grasping abilities significantly enhance children’s interaction with and knowledge of the external environment (e.g., [7,8]). For example, focusing on distinct attributes of an object while manipulating it helps infants associate specific meanings with particular referents during the lexical acquisition process. Thus, when infants reach for and grasp an object, they gather information from the environment and learn to modulate their movements, which gradually become more accurate and oriented towards a specific goal [9]. Relatedly, the ability to plan movements in specific situations allows children to move and orient themselves in the external environment in order to interact with objects and people in a functional way. These interactions with objects are not merely about physical handling but involve complex sensory experiences that integrate visual, tactile, and proprioceptive information. By examining how infants explore and manipulate objects, it is possible to gain valuable understanding of their developmental trajectory in sensory and motor domains [10,11]. Moreover, object manipulation provides insights into multimodal integration abilities. When infants explore objects, they integrate information from different sensory modalities to understand the object’s properties. For example, an infant might visually inspect a toy while simultaneously feeling its texture and weight. This integration is essential for developing a coherent perception of the world and for learning how different sensory inputs relate to one another.

Therefore, manual exploratory actions generate new information for the sensorimotor system that subsequently fosters progress in higher-level cognition. In this regard, studies have demonstrated a solid link between exploratory abilities and improvements in object individuation (e.g., [12]), problem solving [13], and social understanding [14]. Moreover, it has been hypothesized that the interaction with objects enhances communication abilities, including the vocalization production during object exploration [15,16]. 

One implication of the above findings is that delays in the development of these skills may limit interactions between the child and the environment, potentially leading to delays in the acquisition of skills in other domains as well [17]. Mounting evidence highlights a potential connection between motor difficulties and neurodevelopmental disorders, in particular in autism spectrum disorder (ASD; e.g., [18,19,20]; see also [21] for a review). Evidence has confirmed that delays in the motor domain can also have cascading effects in areas that are crucial for ASD, namely social and language development (e.g., [17,22]), leading researchers to highlight motor challenges as a relevant feature in the behavioral profile of ASD (e.g., [23]) and suggesting that those skills could provide significant insights for the early detection of possible ASD. 

While ASD is typically diagnosed in the second–third year of life based on social and communicative criteria, motor skills are typically acquired earlier, and could provide the basis of identification of early markers of ASD (for a review see [21]). For this reason, a growing body of work has started to focus on the younger siblings of children who have already received an ASD diagnosis, as they are considered at elevated likelihood (EL) for ASD compared to infants with no family history of the disorder (typical likelihood, TL; [24]). In this growing field of research, several studies have addressed early motor skills in EL infants and review articles have been published that compared motor development in neurotypical infants and infants with emerging ASD (e.g., [25,26]). In this paper, we aim to contribute to this discussion by providing a theoretical review that closely examines the literature on object exploration and manipulation skills in EL and TL infants. 

The paper is organized as follows. We begin by providing an overview of what is known about fine motor skills during object manipulation tasks in neurotypically developing children (Section 2). Next, we review the major findings on object manipulation and exploration in infants at elevated likelihood (EL) for ASD (Section 3), focusing especially on manual skills (Section 3.1), cascading effects on motor planning abilities (Section 3.2), and language (Section 3.3), and, finally, we discuss theoretical and methodological issues arising from the reviewed evidence and outline some directions for future empirical research (Section 4 and Section 5). 

## 2. Object Exploration and Manipulation in Neurotypical Development

Manual exploration of objects can be conceived of as a sequence of ordered motor acts that require optimal hand and finger control allowing for the physical interaction with an object [27,28,29]. Object exploration encompasses a set of fine motor actions, such as fingering, shaking, transferring, and rotating objects. Additionally, abilities such as mouthing and visual attention are considered key to object exploration (e.g., [30]). 

The ability to explore objects emerges at birth and gradually develops in the first two years of life. Evidence revealed that newborns show, already at eight days of life, a pre-reaching approach of the upper limb [31]. As early as two months of age, infants’ arm movements become more coordinated due to increased head control, and from four months, a new developmental phase appears due to the integration of visual motor skills with improved trunk control, which allows a stable base for reaching movements. 

Studies have shown that the increased control of posture, e.g., neck, trunk, and hip control, positively impacts object manipulation, facilitating visual control of the hand moving towards a target and, therefore, helping infants to reach for and grasp objects and to subsequently explore them (e.g., [32,33]). And improved postural control enhances reaching performance. Thus, during the initial phase of its development, reaching movements are discontinuous and jerky, but with the development of postural control, they become smoother and are organized in a single grasping movement [3,34]. Moreover, the ability to sit independently affects the information that infants can access during multisensory exploration of objects [35]. This developmental milestone, characterized by the ability to maintain an upright seated posture without external support, is implicated in shaping infants’ capacity to engage in comprehensive perceptual analyses of objects, encompassing tactile, visual, auditory, and, potentially, other sensory modalities. 

Object manipulation involves more than the progressive acquisition of muscle and arm control; it also requires an understanding of how movements are planned before being performed. Successful object exploration is achieved through planned and organized sequences of movements. These sequences, often called goal-directed actions, require the ability to compose a succession of motor strategies according to the final goal of the action [36]. In other words, children need to plan in advance how to reach for and grasp an object for manipulation. This complex ability seems to appear by the end of the first year when children become able to adapt their grips anticipatorily according to the size [37] or orientation [38] of an object. Around 18 months of age, they start demonstrating the ability to create motor plans. These motor plans go beyond just considering the immediate spatial limitations of a task. Instead, infants at this stage can anticipate and incorporate the requirements of the upcoming actions into their motor planning. In simpler terms, by 18 months, infants show a more advanced ability to think ahead and organize their movements to meet the demands of a task that goes beyond the immediate environment [39]. 

Infants gain understanding of various properties such as texture, shape, size, color, and sound through exploration of objects [40,41,42,43]. For example, research has shown that 3–4-month-old infants who spend more time with objects have better perception of properties such as the boundaries between two close objects than those who spend less time exploring [12]. Engaging directly with objects enhances infants’ cognitive abilities, positively impacting their performance on various cognitive tasks [44,45]. In particular, when infants were asked to retrieve a toy from a container, those who spent more time exploring objects showed higher success rates and utilized a variety of methods to obtain the toy [44].

Several studies focusing on different factors that affect children’s motor performance during manipulation have replicated findings obtained from adults by Marteniuk and collaborators (1987 [46]). Claxton and colleagues (2003 [47]) carried out a study on 10-month-old typically developing infants to determine whether the precision required by the task influences the kinematics of reaching in infants. Comparing the hand speed during reaching movements of infants who had to reach a ball and put it into a tray (non-precision task) or into a tube with a narrow hole (precision task), they found that infants’ reaching movements are affected by task precision demands. Reaching movements were performed more slowly in the precision than in the non-precision task. Similar results were obtained on infants aged 18–21 months [48]. 

Using a different task, Ornokloo and von Hofsten (2006 [49]) investigated how the complexity of the task affects the motor performance of children from 18 to 26 months of age. Children had to grasp objects of different shapes/sections and insert them into appropriate openings. Task difficulty was modulated according to the different transversal sections (circular, squared, rectangular, elliptical, triangular) and the initial orientation of the object with respect to the opening: vertical orientation (simpler) or horizontal orientation (more difficult, as the child has to rotate the shape in order to align its cross-section to the opening). Findings revealed that younger children failed to accomplish the task of grasping and inserting objects as they did not make any adjustment to fit the aperture, while older children accomplished the task, rotating the object when needed. 

More recently, Contaldo et al. (2013 [50]) studied whether the goal of object manipulation affects motor planning. The authors analyzed grasping patterns during play sessions in a naturalistic set-up of children between 9 and 25 months of age, comparing motor actions that lead to a functional goal (i.e., using the object as a tool) with motor actions that lead to exploratory manipulations (e.g., mouthing, shaking, beating, throwing, pushing objects together or moving). The results showed that grasping was more efficient in goal-directed actions than in the exploratory nonfunctional activity, confirming that in infants, motor planning also depends on its goal. Taken together, the above evidence demonstrates that infants’ motor performance during object manipulation, although it generally improves with age, is affected by the precision of the movement, the functional/exploratory nature of the task, and its spatial complexity. 

To summarize, object exploration emerges as a highly multimodal ability that appears at birth and gradually evolves thanks to the development of fine–gross motor skills, such as fingering and rotating objects, as well as cognitive abilities, such as mental imagery and anticipation of the goal. Infants have the opportunity to learn about the characteristics of objects, integrating visual and haptic information, thus laying the basis for object categorization. Moreover, the acquisition of exploratory abilities can initiate a cascade of cognitive, language, and social developments, which, in turn, might drive further progression in motor domains. For these reasons, object exploration and manipulation have recently been considered as a key domain in development, and researchers have investigated exploratory abilities in children with challenges in the social and cognitive domains with the aim of searching for early markers that may appear in motor development earlier than in other areas of development. 

In the next section, we will focus on studies that investigated object manipulation in infants at elevated likelihood (EL) for autism spectrum disorder (ASD) to assess whether findings reveal key differences in the development of exploratory abilities in EL infants and children with respect to infants with no family history of ASD (about 2–19% of EL children receive an ASD diagnosis, compared to 0.6% of TL children [24,51,52,53,54,55]). 

## 3. Object Exploration and Manipulation in Infants at Elevated Likelihood for ASD

Research on elevated likelihood (EL) for ASD infants’ motor development has aimed primarily at searching for early markers that appear in the development of motor skills and that can be useful for diagnosis and for early interventions. Such research is based on evidence demonstrating that EL children, even if they do not receive an ASD diagnosis, display high individual variability in multiple developmental domains [56,57] and that motor delays in EL infants are detectable from the first months of life (e.g., [58,59,60,61,62]). 

Studies on EL children can be divided into three broad groups based on the variable observed (see Table 1). The first group focuses exclusively on skills related to object exploration, such as grasping, manipulating, looking time, and mouthing. Typically, these studies consider children from 6 to 24 months of age and use standardized scales such as the Mullen Scales of Early Learning (MSEL; [63]) or the Peabody Developmental Motor Scales (PDMS; [64]). The second focus is on a specific ability that is crucial for manual exploration, namely motor planning, which is crucial for goal-directed actions. Finally, the third group includes studies that investigate language development in addition to motor skills, aiming at assessing the effect of motor skill development in relation to language development. In this case, children are typically studied longitudinally from the first year to 24 or 36 months of age. We will group studies according to the above distinction, firstly reviewing studies that focus on object exploration and motor planning, and then, reviewing research addressing the relationship between the motor and linguistic domains. 

### 3.1. Object Exploration in EL Infants

A study by Libertus et al. (2014 [66]) is key in the literature on fine motor skills in EL since it pioneeringly demonstrates that differences in the fine motor abilities of EL infants can be observed as early as 6 months of age. Using both the standardized MSEL and a naturalistic free-play task, the study provides critical evidence for weaker fine motor and reduced grasping activity in EL infants compared with TL infants, suggesting that different developmental trajectories of EL infants in the fine motor domain are evident early in life. However, since differences in grasping behavior were no longer significant at 10 months of age, these findings raise the possibility that the observed differences in fine motor abilities may disappear or, at least, cease to be significant after some time. Further studies have confirmed differences in patterns of exploratory behavior in EL children appearing at 6 months of age (though see [82]). For example, Kaur et al., (2015 [27]) studied infants’ object exploration activity at 6, 9, 12, and 15 months. Children were presented with objects with different shapes, textures, and sizes, thus requiring different exploratory behavior. Using a 1 min free exploration task, they found less grasping at 6 months of age and a delayed increase in dropping of objects in the EL infants group compared to TL children (6–9 months in TL, 12 to 15 in EL). Although object affordances influenced exploratory behaviors in both groups, the results indicated that different exploratory behaviors in EL with respect to TL children might be an early indicator of delays in functional and object-appropriate play. 

Several studies have assessed whether differences in object exploration in EL are limited to manipulatory skills or if they are also apparent in non-manual exploratory skills, such as mouthing and visual behavior. Observing 12 month-old infants during a 30 s object exploration task, Ozonoff et al. (2008 [65]) identified and coded eight specific behaviors related to how infants typically and atypically interact with objects. The four behaviors considered to be typical and age-appropriate included shaking, banging, mouthing, and throwing objects. In contrast, the atypical behaviors were spinning, rolling, rotating objects, and engaging in unusual visual exploration. The study’s findings indicated that there were no significant differences between EL and TL infants in three of the four typical object interaction behaviors. By contrast, the EL group displayed significantly more rotating, spinning, and unusual visual exploration of objects. Interestingly, infants later diagnosed with ASD showed high rates of one specific behavior, namely unusual visual exploration of objects, thus suggesting that looking behavior might be a critical variable to monitor in EL infants (similar findings were reported recently by [83]). 

Koterba et al. (2014 [67]) observed infants at 6 and 9 months of age during a play session with two identical rattles, one sounding and one silent. In addition to manual exploration, the authors coded looking time and mouthing activity. Manual exploratory behaviors were generally similar in the two groups, but differences emerged in non-manual exploratory skills. EL infants spent less time mouthing with respect to TL infants, especially at 6 months, suggesting that EL infants exhibit early delays in the visual and oral exploration of objects. In a subsequent study, Northrup et al. (2017 [68]) focused on infants’ interaction with the sounding/non-sounding rattles and showed that infants in both the EL and TL groups adjusted their rattle shaking behavior to changing contingencies (sound/no sound). Ten-month-old TL infants demonstrated generalized knowledge of the affordances offered by rattles, proving that they had a priori expectations for the rattles. On the contrary, EL infants showed no evidence of such a priori expectations; they were able to learn from contingencies at a similar rate to their TL peers, thus suggesting that they may have difficulty applying that knowledge to predict the outcomes of novel interactions. EL infants, and particularly those later diagnosed with ASD, did not appear to generalize their learning from one interaction to the next.

Malachowski and colleagues (2023 [84]) have recently presented a longitudinal retrospective case study comparing motor and object exploration behaviors of an infant later diagnosed with ASD (target infant, T.I.) between 2.5 and 24 months of age, compared to a control infant (C.I.). The study aimed to identify potential early motor and object exploration markers to shed light on the etiology and development of ASD. Participants were assessed at 2.5, 3, 8, and 24 months, and they participated in a structured play session with objects. The authors evaluated four domains such as fine motor skills, manual object exploration, visual orientation, and task imitation. They also used a standardized assessment, such as the Early Motor Questionnaire (a parent report questionnaire; [85]), to evaluate the development over time. The results highlighted significant differences in the four domains, and according to parent reports, fine motor skills at both 3 months and 24 months were significantly lower in the infant later diagnosed with ASD than the control infant. In addition, T.I. engaged less with presented objects both visually and through manual exploration compared to the control infant. By 24 months, he was able to replicate the goals of tasks but did not imitate the experimenter’s specific actions with blocks and tools. 

Another recent work [71] explored the relationship between object play and the intensity of ASD symptoms in young EL children aged 12–18 months and older children aged 24–36 months. The participants were 44 children who were observed in a play session with objects, focusing on the complexity and type of object play; other measurements were involved for ASD symptoms such as the First-Year Inventory [86] and the ADOS-2 [87]. The study highlighted several key points: (i) it found that less complex and more repetitive object play behaviors are associated with higher severity of ASD symptoms in toddlers aged 12–37 months; and (ii) it also revealed that these play behaviors differ between younger (12–18 months) and older (24–36 months) toddlers, indicating developmental changes in play patterns related to ASD symptoms. The research emphasizes the importance of early identification and intervention in children at risk for developmental disorders, suggesting that monitoring object play could aid in early ASD diagnosis and targeted support. Interesting research by Begum Ali and colleagues (2020 [61]) longitudinally examined whether an increased familial likelihood for ASD, compared to a group of infants at familial likelihood for ADHD and a TL infant group, affects the development of midline crossing behaviors in infants during a naturalistic reaching task. The researchers observed infants at 5, 10, and 14 months old as they engaged with toy blocks. The study found that both groups of EL infants (ASD and ADHD) exhibited significantly fewer instances of hands crossing the body midline compared to those with a typical likelihood, at 10 months of age. The EL for ASD and EL for ADHD groups showed similar patterns and the authors suggested that these motor behavior changes are not disorder specific but may represent a transdiagnostic early risk factor. This highlights the importance of examining behaviors across multiple disorders too. 

Taken together, the reviewed evidence suggests that object exploration and manipulation in EL infants follow distinct developmental trajectories from the first months of life, showcasing differences in fine motor skills like grasping and non-manual exploratory behaviors such as looking and mouthing during free-play interactions with various toys. However, questions arise regarding the nature of these differences, questioning whether they might be transient and tend to diminish or decrease during development. Empirical findings indicate that EL infants exhibit diverse fine motor behaviors during object manipulation early in development (see [82]). Additionally, various non-manual exploratory actions appear to be performed differently by EL children. In particular, findings seem to suggest that visual behavior and mouthing are distinguishing features of EL interaction with objects in the first year of life. At the same time, findings also show that EL infants continuously progress in the acquisition of developmental milestones, although at a different pace to TL peers. For instance, as demonstrated by Jarvis and Iverson (2020 [70]), the transition from novice to experienced sitting was associated with enhanced object exploration in EL infants as shown by studies on TL infants (e.g., [32,33]). Therefore, differences between TL and EL infants must be conceived in terms of a more irregular and variable developmental trajectory that might lead to a delayed or slower acquisition of developmental milestones. Finally, findings seem to suggest that fine motor delays are more persistent in EL infants diagnosed later with ASD [62,82]. 

### 3.2. Motor Planning in EL Infants 

Motor planning plays a crucial role in human development and everyday functioning, encompassing a wide range of activities from basic motor skills to complex cognitive tasks. The ability to plan and execute actions efficiently is fundamental to adaptive behavior and the successful exploration of the environment. Typically, 5-month-old infants exhibit a reactive behavior that relies on haptic feedback obtained via contact with the object; by 8 months, infants have begun to rely on visual information to anticipate contact while still reaching [88,89]. Twelve-month-old infants display arm movements and hand grasps that approximate those of adults [90,91], adjusting their reaching in such a way that they anticipate the size, texture, or orientation of objects prior to contact [92].

Individuals with ASD show specific difficulties in the ability to plan actions towards future goals. Because of the internal processing lag of the motor system (about 250–400 ms, [93,94]), carrying out actions on moving objects requires planning movement in an anticipatory manner, i.e., in a predictive rather than reactive way. In fact, if the actor moves in a reactive way, the result will be unsuccessful reaching, leading to difficulties in manipulating and interacting with objects [95,96,97]. Several studies have shown reactive rather than predictive changes in the muscle activity of autistic individuals during load-lifting tasks [96,98] and reduced ability to prospectively plan actions with respect to future goals [95,99]. Below we address the literature on motor planning in EL infants to evaluate whether such deficits in the ability of planning actions also affect EL infants.

The reach-to-grasp movement, in which a hand moves towards the target object to grasp it with the fingers, is a basic motor action that has been widely used as an experimental task to assess motor planning in infants and toddlers. This ability appears very early in infancy and improves throughout development. Ekberg et al. (2016 [72]) used a reach-to-grasp task to assess whether 10-month-old EL infants’ motor behavior is predictive or reactive. Infants were presented with a task requiring them to reach and grasp a ball rolling on a curvilinear path off an inclined tabletop. Analysis focused on the movement performed by infants to catch the ball. If the reaching movement was initiated before the ball entered reaching space, the reach was considered predictive. If the reaching movement was initiated after the ball entered reaching space, this was considered a reactive reach. The results indicated that TL infants reached for moving objects in a predictive manner, while EL infants performed reactive reaches. Similar behavioral profiles were confirmed by Achermann et al. (2020 [77]) who showed delayed movement onset in EL with respect to TL infants. Moreover, using three-dimensional (3D) motion capture technology to investigate subtle kinematic variables, finer analysis of sub-movements suggested group differences in motor execution and planning abilities, revealing lower peak velocity (i.e., the maximum velocity measured in the reaching task to catch the ball) in the EL group compared to the TL group. The differences observed may be related to a difficulty in using sensory information to tune action parameters, as suggested by Whyatt and Craig (2013 [100]) for children with ASD. 

Examining visual motor integration in infants at EL for ASD provides an insight into the development of planning abilities. Along these lines, Landa et al. (2016 [73]) compared 6-month-old EL and TL infants’ motor performance during a dynamic, interactive dyadic ball-rolling activity with an experimenter. The task was divided into three phases: “ready”, “set”, “go”, depending on what the children were expected to do. In the “ready” phase, the experimenter bounced the ball attracting the child’s attention. In the “set” phase, the experimenter rolled the ball towards the child. In the “go” phase, the child was expected to approach the ball. While the groups did not differ in the “ready” and “set” phases, EL infants displayed decreased predictive action in response to the approaching ball compared to LR infants. Since both groups showed similar visual attention in the “ready” and “set” phases, differences in anticipatory motor behaviors may reflect differences in the construction of an internal model of visuo-motor coupling. Evidence for the possibility of a weakened visuo-motor coupling comes from observations of 4- to 6-month-old infants later diagnosed with ASD, who showed reduced anticipatory mouth opening when a spoon approached during feeding [101].

The study of movement kinematics has recently been transformed by the availability of wireless, wearable sensors that can provide data on the movement of infants and objects in space. This technology allows the collection of high-frequency data on movement that can be analyzed in many ways. For instance, Focaroli et al. (2016 [74]) compared the performance of EL and TL toddlers in two tasks, each involving two conditions differing in the degree of precision required by the goal action. In the ball task (adapted from [47]), children had to either throw a small ball into a transparent plastic tray or insert the same ball into a plastic tube. In the block task, children had to either throw a block into a large open container or place each of five blocks, one at a time, on a target block to build a tower. The results revealed differences between the TL and EL groups only in the block task, and only in the mean acceleration of reaching, with values significantly lower for EL than for TL children. Specifically, the mean acceleration of reaching refers to the average rate at which infants increase their hand movement speed when reaching for objects. In this context, the results indicate that, specifically in the block task, EL infants displayed significantly lower mean acceleration values compared to TL infants. This suggests that, on average, EL infants exhibited a slower increase in hand movement speed while reaching for objects in comparison to TL infants during this task. 

Taffoni et al. (2019 [76]) observed EL and TL children longitudinally between 14 and 36 months of age. Children were asked to insert blocks of different shapes (cylinder, parallelepiped, triangular prism, hexagonal prism) into a box placed centrally in front of them, and they wore bracelets outfitted with magneto-inertial sensors. The shapes were also instrumented with sensors. More complex shapes were more demanding in terms of task precision, and this was expected to affect variables such as reach duration, place duration, and reach acceleration. However, the results revealed no significant group differences between EL and TL children on any of the variables considered. Rather, the kinematics of the reaching phase were affected by the precision required by the task in both groups. This finding is in line with Sacrey et al. (2018 [75]), who examined reach-to-grasp movements of EL and TL children using the qualitative Skilled Reaching Rating Scale [91]. EL children who were later diagnosed with ASD, defined as the EL-ASD group, received lower total scores on the reach-to-grasp movement and on most subcomponents of movement (i.e., orient, lift, and pronate) compared to other children. However, no differences were found between EL children with no ASD diagnosis and TL comparison children. These findings confirm motor planning delays in children with ASD, but also suggest that the reach-to-grasp movement does not show a clearly different developmental trajectory in EL and TL children. 

To summarize, the evidence pointed out some differences in specific aspects of motor planning ability in EL children. When evaluating children of the same age, delayed movement onset and reactive behavior appear to distinguish EL from TL. Specifically, EL individuals and EL-ASD exhibit atypical development in motor planning ability. These findings support the hypothesis that distinctions between TL and EL can be understood in terms of an irregular and variable developmental trajectory, potentially resulting in a delayed or partial acquisition of developmental milestones. On the other hand, it is important to take note that not all studies identified significant differences in motor planning as some research reported similar trajectories of EL and TL children.

### 3.3. Exploratory Abilities and Language Development in EL Infants

The existence of a relationship between exploratory abilities and linguistic development is strongly suggested by several studies showing that exploratory actions such as mouthing and manipulation are predictors of speech fluency (e.g., [102,103,104]), and gesture use is one of the best predictors of receptive and expressive language skills [105]. For these reasons, in the last decade, researchers have addressed the relationship between object exploration and the development of language in EL infants, aiming at assessing whether differences in early motor development might predict poor language abilities at 24–36 months of age. 

Several studies have shown that EL infants exhibit delays in expressive language, and that such delays are predicted by early motor delays in grasping and object manipulation skills. LeBarton and Iverson (2013 [78]) used different measures for assessing motor and language development in children aged between 12 and 36 months old, such as the Infant Oral and Manual Interview (IOM; [102]), the MacArthur-Bates Communicative Development Inventories (CDIs; [106]), and the Mullen Scales of Early Learning (MSEL; [63]). In line with the previous literature, the results confirmed the existence of fine motor delays in EL infants in the first 18 months of life. However, such delays tended to disappear at 24 months in EL infants who did not receive a diagnosis of ASD. CDI scores showed that EL infants exhibited delays in language development at 36 months. Interestingly, the observed differences in fine motor development between 12 and 24 months were related to individual differences in later expressive language at 36 months. Similar findings were obtained by LeBarton and Landa (2019 [81]), who used the Peabody Developmental Motor Scales-2 (PDMS-2; [61]) to test infant motor skills at 6 months of age as well as the Mullen Scales of Early Learning as a measure of general developmental level. The results confirmed that difficulties in grasping and object manipulation and in visual motor integration characterized the ASD and EL-no ASD groups relative to TL infants. Moreover, regarding language, PDMS-2 scores on the Stationary and Grasping subscales predicted expressive language at 30- and 36 months.

Assuming, as suggested above, that EL infants’ motor development follows a different trajectory, one might ask whether something similar happens to language development. The study by Landa and Garrett-Mayer (2006 [82]) is the first prospective, longitudinal study of motor and linguistic developmental trajectories in EL (later diagnosed with ASD or with language delay) vs. TL infants from 6 to 24 months of age. The results showed significant differences in motor skills at 6 months between the EL and TL infants. Participants were classified into three categories—unaffected, ASD, or language delayed (LD)—based on language test scores, the Autism Diagnostic Observation Schedule, and clinical judgment at 24 months of age. At 6 months, no significant differences were observed between groups. However, by the age of 14 months, the ASD group exhibited significantly poorer performance than the unaffected group across all scales except Visual Reception. By 24 months, the ASD group performed notably lower than the unaffected group across all domains, and the language-delayed group in the Gross Motor, Fine Motor, and Receptive Language domains. The developmental trajectory of the ASD group was slower than that of the other groups, demonstrating a significant decline in development between the first and second year. 

Delving deeper into the relationship between early motor skills and the rate of language and communication development, Leonard et al. (2015 [78]) administered the Vineland Adaptive Behavior Scales at 7, 14, 24, and 36 months and the MSEL at 7 months. They found that the rate of expressive language development can be predicted by early motor skill, and that the relationship between motor and language development differs between EL and TL infants. However, in contrast with Landa and Garrett-Mayer (2006 [82]), no significant relationship was found between gross or fine motor skills and the rate of receptive language development in any group. Similar results were obtained by Choi et al. (2018 [80]) on a sample that included EL infants without ASD diagnoses, EL infants later diagnosed with ASD, and TL infants. They analyzed fine motor skills at 6, 12, 18, and 24 months, correlating these scores with expressive language abilities at 36 months, utilizing the MSEL scale for assessment. The results show that EL infants later diagnosed with ASD showed significantly slower growth in fine motor skills between 6 and 24 months, compared to TL infants. Moreover, early fine motor skills were associated with subsequent expressive language skills at 36 months in all three groups (see also [69], for a study on the correlation between action production and word comprehension in EL from 10 to 36 months). 

Together with other studies (e.g., [56]), the results from the above studies suggest that expressive language development is related to early fine motor skills in EL, while a less solid relationship emerges between receptive language and motor skill development. (This finding is in contrast with Toth et al. (2007 [107]), who revealed a significant difference between EL and TL in receptive language and not in mean expressive language ability. Nonetheless, factors related to sample size and statistical analysis might play a role in Toth et al. (2007 [107]). For example, although the overall mean scores of EL in [107] fell within the average range for both groups in expressive language scores, 20% of EL had below average scores.) Based on these results, one might observe that approaching EL vs. TL atypical language development in terms of trajectories might result in a more powerful predictive power. For example, assuming that deficits increase with age, we would expect differences in comprehension between EL and TL children to be detectable earlier than differences in expression, similar to what is found in children with ASD (e.g., [82,108]). 

## 4. Discussion

Over the last decades, empirical research has devoted increasing attention to the development of the motor domain, demonstrating the intimate relationship between motor and other non-motor domains [109,110], such as social and linguistic, that are crucial for human development. Studies show that gross and fine motor skills are positively correlated with linguistic and social capabilities in young children [4,111,112]. At the same time, early motor delays can have cascading effects that go beyond the motor domain [22,113,114]. Several studies have shown that impaired or delayed motor abilities might limit how infants engage with their surroundings and learn through their own actions and experiences (e.g., [115]). 

Based on evidence demonstrating that motor delays in EL infants are evident from the first months of life (e.g., [58,59]), researchers have investigated EL infants’ object exploration, especially manual and non-manual behaviors, fine motor skills, visual attention, and mouthing. Findings seem to converge on the identification of unusual visual behavior and mouthing as distinguishing features of EL infants’ interaction with objects throughout the first year of life. The process of combining information from different sensory modalities, as the motor coordination, is essential for engaging with the environment and in object manipulation; a deficit in multimodal integration and coordination can hinder the ability of EL children to explore and manipulate objects effectively, impacting their overall development [116]. These difficulties can lead to challenges in object exploration, as infants may struggle to synchronize visual, auditory, and tactile information [117] causing behaviors that appear repetitive or stereotyped. At the same time, the results also show that EL infants progress in the acquisition of developmental milestones, although at a different pace compared to TL peers.

Studies focusing on the effects of motor delays on language development suggest that expressive language, rather than receptive, is related to early fine motor skills in EL [115]. Finally, atypical traits in motor planning abilities emerge from studies on reach-to-grasp in EL infants, who tend to show less reactive motor behavior with respect to their TL peers. 

Although significant differences between EL and TL infants may emerge regarding specific variables or aspects of motor abilities at certain developmental stages, the reviewed evidence tentatively concludes that the developmental trajectories of EL and TL infants are more similar to each other than to those of EL-ASD children, whose motor delays are persistent throughout development. At the same time, however, these subtle developmental differences are important to consider. Motor development allows children to gain independence in movement, and it also creates opportunities for knowledge of space and objects, and of oneself as different from the rest of the environment and in visuo-spatial perception. A child who manipulates objects and explores them not only acquires information about different shapes and materials but also creates opportunities to learn sounds and words, thus scaffolding the development of multisensory integration. Showing or sharing an object to a caregiver creates an opportunity in which children are exposed to the verbal label associated with it; the sustained attention in the activity with objects also has important effects on cognitive development. Considering the findings reported above from a developmental cascading effects perspective enables us to contemplate the possible broad impacts of little early difficulties and delays between developmental domains and on the child’s surroundings [114]. Understanding the significant role that motor functions can play in social dysfunctions can enhance our understanding of the disorder’s characteristics. Despite the strong predictive value of early motor symptoms for later ASD symptom severity, this area remains largely under-researched and inadequately addressed [21,118,119]. Moreover, exploring the relationship between motor dysfunctions and other developmental aspects in EL children can lead to the emergence of new intervention approaches. 

That being said, however, language development delays or difficulties may not necessarily be coupled with significant motor deficits, and vice versa [120]. Both domains involve intricate neural processes influenced by several factors, including genetic, environmental, and individual. The discrepancies noted in some studies within this review (e.g., [82] vs. [79,80]) highlight the need to further investigate the moderating factors that may have contributed to the varied findings.

### Future Directions

Finally, to date, studies about EL children present some limitations: (i) many studies have small, non-diverse sample sizes, making it challenging to generalize the findings to the broader population, as suggested in a recent review of Dawson et al. (2023 [121]). (ii) Studies focus mainly from birth to 3 years of age and very little is known beyond that age range. Many studies primarily focus on early motor development and do not extend follow-up assessments into later childhood or adolescence. Long-term outcome data are essential for understanding the persistence, trajectory, and functional implications of motor skill differences in EL infants. It would be important to investigate, for example, how some important skills develop during preschool age, such as, for example, the basic prerequisites for the acquisition of reading–writing and mathematical computation. Indeed, we know that underlying these learnings are phonological, linguistic, fine motor, and visuo-spatial skills. (iii) Studies have carried out various approaches such as standardized assessments, observational techniques, and advanced technological tools, providing critical insights into infant development and ASD risk factors, and offering comprehensive data from different angles. On the other hand, for clinical evaluations and diagnoses, it is crucial to have standardized and validated tools to assess fine motor skills as biomarkers for conditions such as ASD. Accurate measurement and interpretation of infants’ fine motor skills are challenging without such tools. For example, many studies use standardized tests that identify whether specific motor behaviors allow quantifying abilities and/or assessing the presence of deficits relative to the population of the same age. As suggested by Iverson and colleagues (2019 [122]), it could also be useful to analyze the structure, the quality, the frequency, and the usage of a fine motor behavior; this may offer more valuable insights into motor development profiles and with respect to the quality of the motor act. This also highlights the necessity for ongoing research and interdisciplinary collaboration in this domain. (iv) Another important priority for future research is to distinguish the early behavioral patterns among children with ASD from those with other neurodevelopmental disorders. ASD often presents alongside other comorbid conditions (2020 [123]), which can manifest early in development, sometimes even preceding or overlapping with ASD symptoms. For instance, conditions such as developmental coordination disorder (DCD) [124], attention-deficit/hyperactivity disorder (ADHD) [125], and intellectual disabilities may co-occur with ASD [126,127] and impact fine motor skills. Understanding these comorbidities is essential for accurate assessment and intervention. As reported by Begum Ali (2020 [61]), the same behavioral patterns were observed in both EL for ASD and EL for ADHD groups, indicating that subtle motor behavior changes are not necessarily exclusive to one disorder. This underscores the importance of analyzing behaviors across various disorders, highlighting the importance of a multidisciplinary approach to early developmental screening and diagnosis, involving professionals from various fields. Therefore, it would be useful to investigate how the motor behavior of children with biological risk for ASD differs not only from a control group with typical development, but also from groups with atypical development not associated with autism, in order to better differentiate the motor profile of the disorder. Since the transversality of these motor difficulties in several disorders, a careful analysis of control processes to find those specificities typical of the syndrome could allow the diagnostic criteria to be modified by including in the criteria only those motor components specific to the syndrome.

Addressing these limitations requires interdisciplinary collaboration, larger and more diverse samples, longitudinal study designs to track developmental trajectories over time, refined measurement tools, and integration of neurobiological and behavioral assessments. Longitudinal data help in identifying early indicators that might be predictive of later ASD diagnoses, understanding when those indicators are most evident, and providing long-term outcomes for children diagnosed with ASD. This perspective is also essential for evaluating the effectiveness of early detection and intervention strategies. 

By overcoming these challenges, future research can provide a more comprehensive understanding of motor development in infants at elevated likelihood for ASD and facilitate the development of targeted interventions to support optimal developmental outcomes. 

## 5. Conclusions

The present review explored the developmental trajectory of object exploration and manipulation in neurotypical infants and those at elevated likelihood (EL) for autism spectrum disorder (ASD). We aimed to elucidate the emergence, progression, and potential differences in these abilities, shedding light on their implications for broader developmental domains. Research on EL infants has focused on identifying early markers for ASD diagnosis, recognizing that motor delays often manifest in the first months of life. Findings revealed distinct developmental trajectories in EL infants, marked by differences in fine motor skills and exploratory behaviors compared to typically developing (TL) infants. Recognizing and addressing these nuanced motor developmental differences in EL infants are essential, with implications extending into cognitive, communicative–linguistic, and social development. As research continues to unveil the intricate interplay between motor and non-motor domains, ongoing exploration promises to refine our understanding of developmental trajectories, ultimately informing interventions that optimize developmental outcomes for EL children.

## Figures and Tables

**Table 1:** The list of studies included in this review and grouped according to the variables they consider.

Ref.	Year	Authors	Number of Subjects	Age	Topic
[65]	2008	Ozonoff et al.	66 (35 EL)	12–26 months	Object exploration
[66]	2014	Libertus et al.	129 (107 EL)	6–36 months	
[67]	2014	Koterba et al.	31 (15 EL)	6–9 months	
[68]	2017	Northrup et al.	56 (39 EL)	6, 10 months	
[69]	2018	Sparaci et al.	41 EL	10–24 months	
[70]	2020	Jarvis et al.	42 (19 EL)	2.5–36 months	
[71]	2023	Kawa	44 (EL)	13–37 months	
[27]	2015	Kaur et al.	32 (16 EL)	6–16 months	
[61]	2020	Begum Ali et al.	81 (52 EL)	5–14 months	
[72]	2016	Ekberg et al.	45 (29 EL)	10 months	Motor planning
[73]	2016	Landa et al.	107 (66 EL)	6, 14 months	
[74]	2016	Focaroli et al.	29 (15 EL)	18–36 months	
[75]	2018	Sacrey et al.	30 (20 EL)	6–36 months	
[76]	2019	Taffoni et al.	33 (19 EL)	14–36 months	
[77]	2020	Achermann et al.	58 (39 EL)	10, 24 months	
[78]	2013	LeBarton et al.	59 (34 EL)	12–36 months	Exploratory abilities and language development
[79]	2015	Leonard et al.	20 EL	9–40 months	
[80]	2018	Choi et al.	170 (101 EL)	6–24 months	
[81]	2019	LeBarton et al.	140 (89 EL)	6–36 months	
[82]	2006	Landa et al.	87 (60 EL)	6–24 months	
